# Adiposity and changes in movement-related behaviors in older adult women in the context of the built environment: a protocol for a prospective cohort study

**DOI:** 10.1186/s12889-019-7905-8

**Published:** 2019-11-14

**Authors:** Roman Cuberek, Jana Pelclová, Aleš Gába, Jana Pechová, Zuzana Svozilová, Miroslava Přidalová, Nikola Štefelová, Karel Hron

**Affiliations:** 1 Institute of active lifestyle, Faculty of Physical Culture, Palacký University Olomouc, třída Míru 117, 771 11 Olomouc, Czech Republic; 2 Department of Mathematical Analysis and Applications of Mathematics, Faculty of Science, 17. listopadu 1192/12, 771 46 Olomouc, Czech Republic

**Keywords:** Causality, Healthy aging, Health risk behaviors, Risk factors, Regression analysis

## Abstract

**Background:**

In older adults, sedentary behaviors increase while physical activity decreases over time following the compositional nature of 24-h behaviors. These changes in movement-related behaviors (MRBs) might be associated with unhealthy weight gain and several health comorbidities. However, information is lacking on how obesity influences longitudinal changes in the composition of MRBs in older adults. Furthermore, the moderating effect of the built environment on prospective associations between obesity and MRBs in older adults is not fully understood. Therefore, using an integrated time-use approach, this study aims to identify prospective associations between obesity and MRBs together with an assessment of the moderating effect of the built environment in elderly women.

**Methods:**

The study was designed as a prospective 7-year follow-up study. It is based on two previous cross-sectional studies that enable the use of participant data (women aged 60+ years, *n* = 409) as a baseline dataset in the current study. All methods designed for 7-year follow-up are based on previous studies. The data collection comprises device-based measurement of MRBs (ActiGraph GT1M accelerometer), objective assessment of body adiposity (multi-frequency bioelectrical impedance analysis), subjective assessment of the built environment (NEWS-A questionnaire), and other possible confounding factors. Time spent in sedentary behavior, light physical activity, and moderate-to-vigorous physical activity will be used as three components in a composition reflecting individual MRBs. In linear multiple compositional regression analysis assessing the prospective association between obesity and MRBs, the 7-year follow-up composition of the three mentioned components represents the dependent variable. The 7-year changes in the percentage of body fat (body adiposity), baseline composition of MRBs, and parameters of the built environment represent regressors.

**Discussion:**

This study will use an integrated time-use approach to explore causality from obesity to device-measured behaviors in older women. The design and respective analysis consider the compositional nature of MRBs data and the potential moderating effects of various factors. A comprehensive assessment of causality may help to develop multilevel interventional models that enhance physical activity in older adults.

## Background

The world’s population is aging [1], and this process is accompanied by the loss or reduction of health-enhancing physical activity (PA) and the concurrent rise of sedentary time [2]. At the same time, the proportion of older adults who are overweight or obese increases [3]. These issues have resulted in an urgent need for effective social, educational, medical, environmental and economic solutions to mitigate these negative trends.

In the older adult population, a moderate amount of regular PA can minimize the physiological effects of a lifestyle that is generally sedentary and increase active life expectancy by limiting the development and progression of chronic diseases and disabling conditions [4], thus reducing medical and social services costs [5].

Obesity has been found to be significantly inversely associated with PA in numerous studies [6]. Moreover, other studies in which participants were segmented into multiple groups according to the volume or intensity of their PA and incremental differences in BMI or adiposity parameters supported a dose-response relationship [7, 8]. However, these cross-sectional studies did not allow for conclusions about causality to be drawn.

A theory-based study in an adult population using the theory of planned behavior [7] confirmed a one-directional relationship and suggested that obesity could be a direct predictor of future PA. A number of prospectively designed studies concluded that obesity is a risk factor for a decrease in PA [9–11], an increase in physical inactivity [9, 12], and a sedentary lifestyle [13, 14] in the future. However, the majority of the abovementioned studies focused on middle-aged populations, with the rare inclusion of older adults [14], and have often been limited by the use of self-reported data of MRBs and obesity status. The most recent findings [15], which indicate approximately twofold stronger associations between PA and adiposity as measured with objective measurements than with self-reported PA, emphasize the need to incorporate objective measures in future studies.

These assessment methods, while typical for large-scale studies, are prone to increasing measurement error and misclassification [16, 17]. Thus, a prospectively designed investigation examining the association between obesity and PA level using device-based methods, including accelerometer-based measures of PA and a well-validated body composition assessment in an old-aged population, which would likely yield valuable valid data, might help to provide more valuable insights.

Social ecological models [18, 19] suggested that specific health-related behaviors (e.g., regular moderate PA) are influenced at multiple levels, ranging from policy and the built environment to psychosocial and individual factors. The influence of environmental exposures on individual health behaviors may increase with age, as older adults spend longer periods of time in or near residential areas. As adults age and increasingly lose control of their functioning, they become more vulnerable to environmental challenges and sensitive to environmental constraints [20–22]. The majority of previous studies have indicated a positive association between the quality of the neighborhood environment and PA in older adults [22, 23]. In the Central European population aged 50+ years, neighborhood aesthetics, land use mix-proximity, street connectivity and neighborhood safety were found to be associated with higher odds of achieving health-enhancing PA [24, 25]. The built environment-obesity relationship is not as clear in older adults [23]. Some studies have indicated that neighborhood walkability (i.e., describing the extent to which the environment is conducive to walking and an active lifestyle) and land use mix are negatively associated with obesity [22, 26]. However, some studies have indicated a null association between certain characteristics of the built environment and obesity [27, 28]. In Central Europe, obesity was found to be lower in older women living in highly walkable areas [29].

Obesity in older age could be a possible barrier to involvement in regular PA [30]. Examining the contribution of obesity to changes in future activity levels is desirable and could lead to cues for predicting PA decreases in old age years before this behavior manifests. Such findings may also serve to create more specific programs and health promotion strategies to prevent weight gain and to avoid decline in habitual PA late in life. Elderly women are an important part of the target group for PA promotion, since the menopausal transition years place them at high risk of abdominal obesity [31], which is a widely recognized risk factor for cardiovascular and metabolic diseases [32]. Since multilevel interventions may be the most effective way to increase PA in older adults [33], studies should shift from simplistic to more complex models that take potential moderators and mediators into account [34]. Accounting for the moderating effect of certain environments might be appropriate, since more walkable environments may help older adults with obesity to engage in more PA. It is necessary to describe the role of the built environment in the long-term influence of obesity on PA in older adults to guide the development of environmental and policy initiatives that might lead to the development of both physical-activity-friendly and aging-friendly neighborhoods.

Recent epidemiological studies highlight the fact that behavior should be seen as a time-use variable in the finite number of discrete behavior modes that are changing. In this approach, any behavior can be understood as a varying time use in concrete patterns of behavior. Consequently, movement-related behavior (MRB) data should be considered compositional, and an appropriate methodological approach has to be applied in corresponding studies [35, 36]. In this regard, based on energy consumption, body position, and consciousness, studies commonly differentiate among four discrete composite parts of 24-h units: time spent in sedentary behavior (SB), time spent in light PA (LPA), time spent in moderate-to-vigorous PA (MVPA), and time spent in sleep [36].

Recently, Pedišić, Dumuid and Olds [37] noted that the application of inappropriate methods in compositional data can lead to incorrect conclusions. Above all, they described the risks related to the application of regression analysis in previous epidemiological studies in which the compositional character of data is not respected. Consequently, they also presented a proper statistical data procedure applicable in MRB data. Their approach is based on a statistical compositional data analysis [38]. We can therefore register a number of epidemiological studies in the last few years applying this new statistical approach that respects the compositional character of MRB data. We assume that this integrated time-use approach [37] has the potential to provide new insight into knowledge about the risk of obesity with respect to MRBs.

### Aim

The primary aim of the study was to use an integrated time-use approach to investigate the prospective associations between adiposity and device-measured MRBs in older adult women. The specific aims are as follows:

1. To describe the longitudinal changes in daily MRBs (three-part composition consisting of SB, LPA, and MVPA) in older adult women.
2. To describe the longitudinal changes in adiposity in older adult women.
3. To investigate the prospective associations between changes in adiposity and MRBs in older adult women.
4. To examine the prospective association between adiposity and the longitudinal reallocation of time spent in particular components of MRBs in older adult women.
5. To investigate the moderating effect of the built environment on prospective associations between adiposity and MRBs in older adult women.

### Hypotheses

Our central hypothesis is that obesity prospectively leads to changes in the composition of time spent in various types of MRBs in elderly women. More specifically, these changes are assumed to be typically based on an increase in SB at the expense of other more intense PA. Another study hypothesis is that the built environment moderates a prospective association between obesity and MRBs in elderly women.

## Methods/design

### Study design

**Fig. 1:**
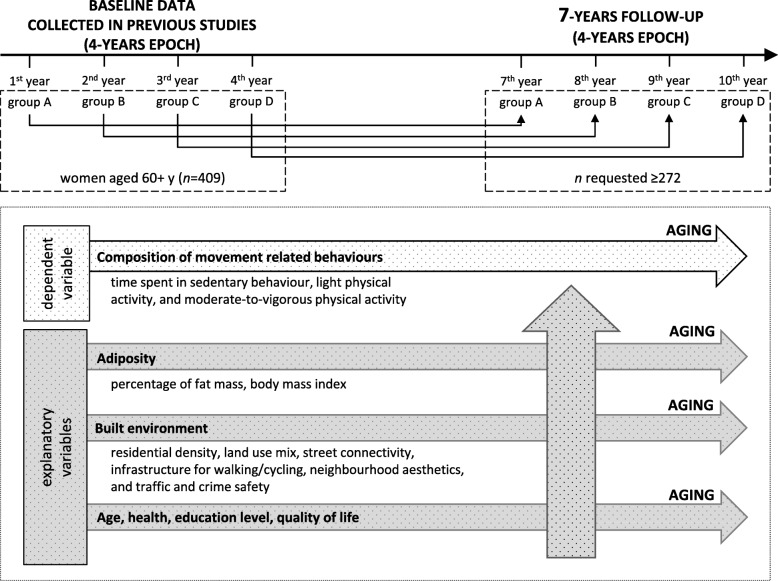
Flow chart of the study

The prospective cohort study is based on two previous cross-sectional studies [8, 39] originating from the institution of the authors. With the agreement of all original authors, we are using data as a baseline dataset for current study purposes. In the mentioned studies, data collection lasted for 4 years. We designed a 7-year prospective follow-up. Therefore, the exact term of follow-up data collection will be individually tailored to reach the 7-year period in each of the participants. We consider a 7-year period to be sufficient for hypothesis verification. The design of the study is depicted in Fig. 1.

According to measurements in the previous studies [8, 39], which provide baseline data, all procedures of data collection (device settings, process and measurement conditions) will be respected in the follow-up.

A measurement of time spent in SB, LPA, and MVPA is based on accelerometry. According to Freedson’s cut-off points [40], each minute of wearing time with < 100, 100–1951, and ≥ 1952 counts per minute are considered to be SB, LPA, and MVPA, respectively. Body fat percentage (FM%) will be classified as ‘normal‘ (≤35%) and ‘obese’ (> 35%) [41].

The prospective association between obesity and MRBs might be affected by other possible factors. Hence, additional information from participants regarding demographic (age, employment status) [42], socioeconomic (education) [43, 44], health (self-reported health) [45], and behavioral (smoking status) [46] factors is obtained through a questionnaire. Self-reported health is assessed via a simple question that asks about the current health of the respondents; the responses were classified as “excellent”, “very good”, “good”, “fair” or “poor”.

For baseline and follow-up data, the daily composition will consist of three components of waking MRBs (SB, LPA, and MVPA). Compositional analysis takes into account the relative scale of the data by focusing on the log-ratio between parts. This approach ensures that it does not matter if only a subset of components is available for the analysis instead of the whole composition [47]. In this study, the data will be close to 16 h for the purpose of isotemporal substitution modeling [48], which represents an ideal active portion of the day (assuming 8 h of sleep a day).

### Sample size and power calculation

Employing the power calculation for linear multiple regression, we determined the minimal detectable effect size to be 6.3%. The principal calculation is based on significance level = 0.05, power = 0.80, number of predictors = 12 (number of observed outcomes that are expected to be included in model), sample size of baseline dataset = 409 and estimate of dropout in the 7-year follow-up = 30% (follow-up sample size ≈ 287 participants).

### Participants

The baseline dataset includes healthy women aged 60+ years who completed their education and PA programs at the University of the Third Age. The exclusion criteria for baseline study involvement were the inability to walk without any prosthetic aids and being in residential care. The merged samples from the studies allow us to use data from 409 women; 313 of them were classified as ‘active’, and of these women, 134 were classified as ‘obese’. Detailed demographic characteristics and the process of recruitment are described in two of the above-mentioned cross-sectional studies [8, 39]. Within these studies, the participants were asked about their willingness to participate in future research. In case of an agreement, they provided written consent.

### Data collection

#### Movement-related behaviors

In accordance with the baseline studies, the ActiGraph GT1M accelerometer device (Manufacturing Technology Inc., FL, USA) will be used to record counts per day for eight consecutive days during waking hours but not during bathing or swimming activities. The research staff will personally check the fastening of the device at the right hip. The accelerometers record activity counts in one-minute periods. Nonwear time is considered to be a period of 60 consecutive minutes in which no movement was detected (0 counts per minute), allowing for 2 min of interruptions > 0 counts per minute. This algorithm is provided in the manufacture’s software (ActiGraph, LLC., Pensacola, FL, USA). For the assessment of accelerometer-derived SB, LPA, and MVPA, a ‘valid day’ will be defined as one in which the participant accumulated ≥ 10 valid hours of wear time. Only the participants providing valid data for at least 4 days (3 workdays and 1 weekend day) will be included in the analyses.

#### Adiposity

In accordance with the abovementioned original studies, body height will be measured while participants are barefoot using a P-375 portable anthropometer to the nearest 0.1 cm (Trystom, Olomouc, Czech Republic). Multifrequency bioelectrical impedance analysis (InBody 720 device, Biospace Co., Ltd., Seoul, Korea) will be used to measure body weight (to the nearest 0.1 kg), or FM%. Recently, the procedure to measure adiposity using the InBody 720 device has been validated in elderly women [49].

#### Built environment measurement

In accordance with the abovementioned original studies, a modified and culturally adapted abbreviated version of the questionnaire titled ‘Neighborhood Environment Walkability Scale’ (NEWS-A) [50] will be used to obtain information about the perceived built environment. The NEWS-A is an instrument that assesses the perception of neighborhood design features related to PA, including residential density, land use mix (including both indices of proximity and accessibility), street connectivity, infrastructure for walking/cycling, neighborhood aesthetics, and traffic and crime safety.

### Statistical analyses

Statistical analysis and data management will be undertaken using SPSS version 22.0 software (SPSS, Chicago, IL, USA) and R 3.4.2 software (R Foundation for Statistical Computing, Vienna, Austria). Imputation of missing values (using the model-based imputation adapted also for dealing with compositional data from the R-package robCompositions) is considered.

Compositional descriptive statistics, including compositional geometric means (central tendency) and a variation matrix (dispersion), will be calculated to characterize MRBs and respective changes in a 7-year period. Ternary diagrams will be used to depict prospective associations between obesity and MRBs.

Compositional linear multiple regression will be used to assess prospective associations between adiposity and the composition of MRBs. We will use robust compositional regression to avoid the influence of possible outlying observations [51]. Alpha was set at 0.05 to determine statistical significance.

## Discussion

During the aging process, older adults become less active and more sedentary, which are considered to be risk factors for mortality. The time spent in different MRBs throughout the day might be associated with body composition. An inverse association between obesity and PA volume and positive associations between obesity and SB were found in cross-sectional [6] and prospective studies [12–14]. However, these studies focused mainly on middle-aged populations with the inclusion of mainly self-reported assessment methods. Therefore, our study will mainly focus on older adults, including adults in the transition from age 65+ to age 70 + .

Few studies have suggested that obesity may lead to a subsequent increase in SB among middle-aged and older adults [14, 52]. However, these studies considered SB to be an independent predictor of obesity without conceptualizing it as a part of the time-use composition.

Studies using an integrated time-use approach to explore the associations between device-measured MRBs and obesity among older adults have, to our knowledge, all been cross-sectional [52–56]. Evidence from these studies appears to consistently suggest that reallocating sedentary time to PA may be associated with reduced adiposity, with the magnitude of associations being greater for higher intensities of PA [56]. However, there is a lack of evidence linking changes in obesity and MRBs over time, although such evidence could underpin obesity interventions and public health policy for older people.

In general, it seems that the strongest association with obesity is observed when time is reallocated from SB to MVPA [55], but reverse causation is also plausible. Studies using an integrated time-use approach to explore the associations between device-measured MRB and obesity among older adults have, to our knowledge, all been cross-sectional [52–56]. Evidence from these studies appears to consistently suggest that reallocating the time spent in SB to the time spent in PA may be associated with reduced adiposity, with the magnitude of associations being greater for higher intensities of PA [56]. However, there is a lack of evidence linking changes in obesity and MRBs over time, although such evidence could underpin obesity interventions and public health policy for older people.

In general, it seems that the strongest association with obesity is observed when time is reallocated from SB to MVPA [55], but reverse causation is also plausible. Especially in older adults, increasing adiposity might lead to PA reduction with the concurrent rise in sedentary time. However, evidence about the influence of obesity and changes in obesity on the later composition of MRBs in older age is missing. Therefore, the major strength of this study is that it will use an integrated time-use approach to investigate how obesity is prospectively associated with longitudinal changes in the time spent in various MRBs.

The built environment might have a powerful impact on the health of older people and on their ability to successfully age in place [57]. Although obesity cannot be directly affected by the built environment, the built environment clearly has a relationship with obesity as a consequence of PA. A recent review on obesity and the built environment highlighted the lack of longitudinal research on this topic [58]. Hence, another strength of this study is the employment of the built environment as a potential moderating variable affecting longitudinal associations between obesity and MRBs.

The main limitation of the study is that the baseline sample cannot be considered fully representative of older adults. In addition, the sample will include only women; hence, the results cannot be extrapolated to men without caution. Due to the recruitment of volunteers, we may expect selection bias (healthier and more educated sample with fewer smokers compared to the general population) [59]. Moreover, we expect a relatively high dropout of participants aged 70+ years in the 7-year follow-up, which can lead to unspecific changes in the baseline profile of the population.

With an integrated time-use approach, this study is the first longitudinal study investigating the prospective association of obesity and MRBs in older women. The findings from this study might help to clarify the causality of obesity—MRB association, which is necessary for successful weight gain interventions. Moreover, the insights provided into the potential moderating effects of the built environment may help to develop multilevel interventional models in which physical activity in older adults is enhanced.

## Data Availability

The baseline datasets are available from the corresponding author on reasonable request.

## Abbreviations

BMI: Body mass index
FM%: Fat mass percentage
LPA: Light physical activity
MRBs: Movement-related behaviors
MVPA: Moderate-to-vigorous physical activity
PA: Physical activity
SB: Sedentary behavior
